# Integrated transcriptome, metabolome and phytohormone analysis reveals developmental differences between the first and secondary flowering in *Castanea mollissima*

**DOI:** 10.3389/fpls.2023.1145418

**Published:** 2023-03-16

**Authors:** Cai Qin, Tingting Du, Ruiyu Zhang, Qiujie Wang, Yang Liu, Tianyi Wang, Hongyan Cao, Qian Bai, Yu Zhang, Shuchai Su

**Affiliations:** Key Laboratory for Silviculture and Conservation, Ministry of Education, Beijing Forestry University, Beijing, China

**Keywords:** chestnut, secondary flowering, female flower, environment, metabolites, phytohormone

## Abstract

**Introduction:**

Chestnut (*Castanea mollissima* BL.) is an important woody grain, and its flower formation has a significant impact on fruit yield and quality. Some chestnut species in northern China re-flower in the late summer. On the one hand, the second flowering consumes a lot of nutrients in the tree, weakening the tree and thus affecting flowering in the following year. On the other hand, the number of female flowers on a single bearing branch during the second flowering is significantly higher than that of the first flowering, which can bear fruit in bunches. Therefore, these can be used to study the sex differentiation of chestnut.

**Methods:**

In this study, the transcriptomes, metabolomes, and phytohormones of male and female chestnut flowers were determined during spring and late summer. We aimed to understand the developmental differences between the first and secondary flowering stages in chestnuts. We analysed the reasons why the number of female flowers is higher in the secondary flowering than in the first flowering and found ways to increase the number of female flowers or decrease the number of male flowers in chestnuts.

**Results:**

Transcriptome analysis of male and female flowers in different developmental seasons revealed that EREBP-like mainly affected the development of secondary female flowers and HSP20 mainly affected the development of secondary male flowers. KEGG enrichment analysis showed that 147 common differentially-regulated genes were mainly enriched from circadian rhythm-plant, carotenoid biosynthesis, phenylpropanoid biosynthesis, and plant hormone signal transduction pathways. Metabolome analysis showed that the main differentially accumulated metabolites in female flowers were flavonoids and phenolic acids, whereas the main differentially accumulated metabolites in male flowers were lipids, flavonoids, and phenolic acids. These genes and their metabolites are positively correlated with secondary flower formation. Phytohormone analysis showed that abscisic and salicylic acids were negatively correlated with secondary flower formation. MYB305, a candidate gene for sex differentiation in chestnuts, promoted the synthesis of flavonoid substances and thus increased the number of female flowers.

**Discussion:**

We constructed a regulatory network for secondary flower development in chestnuts, which provides a theoretical basis for the reproductive development mechanism of chestnuts. This study has important practical implications for improving chestnut yield and quality.

## Introduction

Secondary flowering is the phenomenon in which a tree blooms more than twice a year. Most plants, such as the apple (*Malus* spp.) (Chitu and Paltineanu, 2020), sweet cherry (*P. avium*) (Fadón et al., 2021), plum (*P. domestica*) (Głowacka et al., 2021), peach (*P. persica*) (Kurokura et al., 2013; Romeu et al., 2014) and other fruit trees of the Rosaceae family, usually bloom once a year in spring. The fruits gradually increase in size and colour and ripen in the summer and autumn. Some plants, such as pear trees (Wei et al., 2022) and magnolias (Xuan et al., 2022), exhibit an anomaly in flowering twice a year under certain conditions. For ornamental plants, the second flowering is due to its extended flowering period and popularity (Iwata et al., 2012; Xuan et al., 2022; Xue et al., 2022); however, for perennial fruit trees, the second flowering and fruiting consume large amounts of nutrients, weakening the tree. The cold resistance of plants during winter is weakened, resulting in a reduced number of flowers and fruit production in the following year (Qiu et al., 2019).

Chestnuts are important non-wood products, and their fruit is edible and rich in nutrients (Carocho et al., 2014; Zhang et al., 2015; Chen et al., 2019; Cheng et al., 2022). However, chestnuts have fewer female flowers than male flowers, and the low yield per unit is a serious constraint to economic development (Chen et al., 2019; Cheng et al., 2022). According to the normal phenological period, the flowering period of chestnuts in northern China is from April to June, whereas some chestnut varieties have abnormal flowering from July to September. For example, ‘Zunhuaduanci’ flowers twice a year and produces two fruits. The formation factors of secondary flowering are complex and uncertain. Current research is only at the physiological level for secondary flowering in chestnut. Thus, due to the lack of relevant genetic information and limited research on the mechanisms of secondary flowering in chestnut, the intrinsic mechanism and root cause of secondary flowering remains unknown.

Once-flowering temperate fruit trees usually bloom in spring (Kurokura et al., 2013). Flower bud differentiation and flowering in fruit trees are complex processes of organ morphogenesis. These processes are the result of joint participation and coordination of various factors within the plant body and the regulation of the tree which is caused by the influence of external environmental factors. Under normal conditions, most flowering plants grow nutritionally in spring, and flower primordia appear in the meristematic tissues of branch tips or lateral buds in summer and autumn. As winter approaches, plants are forced into dormancy due to low temperature stress. Most flowering organs complete differentiation before dormancy and then resume growth and flowering when flower buds develop more rapidly in conjunction with a slow rise in temperature in spring (Amasino, 2010). This cyclic flowering in plants is mainly caused by light and temperature. With the seasonal rotation of external environmental signals, the growth and development of the plant changes in small increments on a daily basis. This variation arises from daily calibration of the endogenous biological clock, a life strategy of plants to cope with the 24-hour circadian rhythm of the Earth, which can enhance environmental adaptation (Ni, 2005; Hotta et al., 2007; Chen et al., 2019). In a photoperiod-dependent flowering induction pathway, changes in day length induce the expression of the flowering integrator *FLOWERING LOCUS T* (*FT*) gene in leaves, and FT proteins are transported to the apical meristem tissue to induce plant flowering (Turck et al., 2008). The abundance of *FT* gene expression depends mainly on the function of the CONSTANS (CO) protein, which acts as a transcriptional activator of the *FT* gene. Biological regulation of CO transcription and the multiple effects of various photoreceptors on changes in CO protein stability are critical for CO protein accumulation under favourable conditions. Expression of *CO* genes is controlled by the biological clock, causing their mRNA abundance to oscillate throughout the day. The *CYCLING DOF FACTOR* (*CDF*) gene, driven by the biological clock, acts as a repressor of flowers and can strongly inhibit *CO* transcription. In the morning, *CDF* expression may be induced by two morning components, CIRCADIAN CLOCK ASSOCIATED 1 (CCA1) and LATE ELONGATED HYPOCOTYL (LHY), which are related MYB transcription factors. In contrast, in the afternoon, their expression appears to be repressed by the action of the PSEUDO RESPONSE REGULATOR (PRR) transcriptional repressor proteins PRR5, PRR7 and PRR9 (Luo et al., 2021; Oravec and Greenham, 2022). In *Arabidopsis*, long daylight stabilizes the CO protein, which induces *FT* gene expression and promotes flowering. However, under short daylight conditions, *FT* gene expression levels are very low, resulting in a delayed flowering time (Niwa et al., 2007; Yeang, 2013). The light environment and temperature are the two main inputs that control the biological clock. The ambient temperature can also induce the circadian rhythm to calibrate flowering time. Low temperatures can induce dormancy. Global warming leads to a significant increase in temperatures from autumn to spring and insufficient cooling in winter, which affects plant dormancy, resulting in flowering disorders in the Japanese pear (*Pyrus pyrifolia*) (Tominaga et al., 2021). In soybean, high temperatures induce the expression of *GmFT2a* and *GmFT5a* as well as a group of *GmCOL* genes to promote flowering (No et al., 2021). In conclusion, flower bud differentiation in different plants is responsive to environmental requirements, and reasonable regulation of the flower bud differentiation range can change the flowering time.

Plants have diverse types of flowering and complex flowering mechanisms, and various endogenous substances and exogenous signals can affect and regulate flowering timing (Srikanth and Schmid, 2011). In addition to the external environment, the metabolite content, hormone levels, and gene expression of trees also affect the flowering processes of plants (Endo et al., 2018). The biological clock and photosynthesis are mutually regulated to protect plants from damage caused by photoinhibition through the regulation of carotenoid biosynthesis. Carotenoids are precursors of abscisic acid (ABA) synthesis, and ABA signalling is regulated by the biological clock (Yari Kamrani et al., 2022). As an inhibitor of flowering in *Arabidopsis*, it may also be involved in the induction of flowering under certain conditions (Riboni et al., 2016; Endo et al., 2018). Ethylene-responsive element-binding protein (ERF) is involved in the regulation of flowering in chrysanthemums, and overexpression of ERF110 can promote floral transition by tuning the circadian clock (Huang et al., 2022). In addition to hormones, lipids can be used as signalling metabolites for flowering time in *Arabidopsis* and oilseed rape. Phenolics and phenylpropanoids are also involved in the regulation of plant flowering (Chakraborty et al., 2022). The expression of genes related to the anthocyanin synthesis pathway, such as *Chalcone synthase (CHS)*, *Chalcone isomerase (CHI)*, and *Dihydroflavonol-4-reductase (DFR)*, under a light/dark cycle exhibits a circadian rhythm (Nguyen and Lee, 2016; Hildreth et al., 2022). In addition, phenylalanine is converted to t-cinnamic acid, and cinnamic acid is converted to cinnamic acid. To induce stress, phenylalanine ammonia lyase (*PAL*) gene expression, PAL enzyme activity, and salicylic acid (SA) content were increased to activate flowering regulation mechanisms (Wada et al., 2014).

The external environment also affects sex differentiation in plants (Talamali et al., 2003). Because the sex of plants is plastic, the external environment can regulate it by affecting gene expression, endogenous hormones, and metabolite content (Endo et al., 2018; Santiago and Sharkey, 2019). High temperatures and long daylight exposure inhibit the formation of female flowers in cucumbers (Lai et al., 2018). However, other studies have shown that male reproductive development is usually more susceptible to high temperatures than pistils and ovules (Santiago and Sharkey, 2019). Sustained heat stress causes cytoplasmic male sterility in soybean, and *HSP20* can act as a protective gene to improve heat tolerance during flowering (Ding et al., 2020). Low temperatures hinder the anther developmental stage in chickpea, causing flowers to abort; however, at room temperature, normal reproductive patterns can subsequently resume (Kiran et al., 2019). Differences in light amount caused sexual instability in *G. sylvaticum* (Varga and Kytöviita, 2016). The higher percentage of blue light in the R2B1 treatment significantly induced the formation and accelerated flowering time of female flowers in cucumber (Song et al., 2018). Therefore, the expression of plant sex is complex and the mechanisms of sex determination are diverse (Stehlik et al., 2008).

In this study, we combined transcriptome sequencing and metabolome analysis to analyse the differences between first and secondary flower development in chestnut, and also identified and evaluated the phytohormone content of male and female chestnut flowers during spring and late summer flowering. The expression patterns of regulatory genes and related metabolites involved in Circadian rhythms-plant, Phytohormone signalling, Flavonoid pathways, and Carotenoid biosynthesis during the development of male and female flowers in different seasons were investigated. Regulatory networks for the key genes and metabolites were constructed. Based on these findings and mechanistic studies, the causes of secondary flower formation in chestnuts were explored for the first time, as well as the key factors for the higher female-to-male ratio of secondary flowers than that of first flowers. The results of this study are expected to provide new ideas for preventing chestnut secondary flowering and regulating the ratio of female to male flowers and provide theoretical support for increasing female production and reducing male production.

## Materials and methods

### Plant materials

The study site is on the Weijinhe Forestry Farm, Zunhua City, Hebei Province, China, between 117°34′–118°14′ E and 39°55′–40°20′ N at 60 m asl. The Chinese chestnut cultivar selected is ‘Zunhuaduanci’, which blooms twice a year after flower bud differentiation in the spring and summer. The trees were 12 years old and full bearing, with robust growth, uniform size, an average height of 3 m, a crown width of 3 m, and a planting density of 3 × 5 m, with normal cultivation management.

In spring, two to three male flower clusters were collected from the base of the male inflorescence when they began to appear on 30 April (FMaM). One to two female flower clusters were collected from the base of the mixed inflorescence (FMiF) when catkins started to appear on 16 May. Samples were taken at 5–7-day intervals until 1 June when sampling ended when the male and female flowers opened. Female and male flowers were mixed separately. Summer flowering began in early July, and male flowers at the base of the male inflorescence in the lower part of the fruiting branches (SMaM) and female flowers at the base of the mixed inflorescence (SMiF) were sampled every 2–3 days. All samples from the four trees were taken as one replicate, and three replicates were used for the RNA-seq, metabolomic, and plant hormone analyses.

### Transcriptome sequencing and analysis

Collected samples were used to extract total RNA from female and male chestnut flowers using the Plant Total RNA Extraction Kit (Promega Biotech Co., Ltd., Beijing, China). Agarose gel electrophoresis was used to analyse RNA integrity, and a Qubit 2.0 fluorometer was used to measure the RNA concentration. Illumina sequencing was performed according to the manufacturer’s protocol at MetWare Biotechnology Company (Wuhan, China). The reference genome is available at http://gigadb.org/dataset/view/id/100643. The data were filtered to obtain clean data, which were compared to a specified reference genome for sequence alignment. Mapped data were then used for structural-level analysis. High-quality clean data were used for all downstream analyses. Expression level analyses, such as differential expression analysis, functional annotation of differentially expressed genes, and functional enrichment, were performed based on the expression of genes in different samples or different sample sets. Fragments per kilobase of transcript per million mapped fragments (FPKM) were used as a measure of transcript or gene expression levels. Differentially expressed genes (DEGs) were screened for |log2FoldChange (FC)|≥1 and false discovery rate (FDR) < 0.05. The R package ‘Phyper’ (www.r-project.org) was used and the Kyoto Encyclopedia of Genes and Genomes (KEGG) analysis was performed with a cut-off value of *P* < 0.05.

### Extensive targeted metabolomic estimation and analysis

Metabolome analysis was performed by the MetWare Biotechnology Company. A total of 100 mg of freeze-dried powder of male and female flowers was dissolved in 1.2 ml 70% methanol solution, vortexed six times for 30 s every 30 min, and kept in a refrigerator at 4°C overnight. Before UPLC-MS/MS analysis, the extracts were centrifuged at 12,000 rpm for 10 min and filtered (SCAA-104, 0.22 µm pore size; ANPEL, Shanghai). A UPLC-ESI-MS/MS system was used to evaluate the sample extracts (UPLC, SHIMADZU Nexera X2, www.shimadzu.com.cn/; MS, Applied Biosystems 4500 Q TRAP, www.appliedbiosystems.com.cn/). The measurements were performed according to the method described by Song et al. (2022). The variable importance in projection (VIP) value was used to assess the effect of the expression pattern of each compound on the classification of each group. A VIP ≥ 1 and a fold-change ≥ 2 or ≤ 0.5 between pairwise comparisons, as well as a *P*-value < 0.05, were used to identify differentially accumulated metabolites (DAMs).

### Detection and analysis of plant hormones

The above samples (50 mg) were weighed, an appropriate amount of internal standard was added, and hormones were extracted using 1 ml of methanol/water/formic acid (15:4:1, v/v/v). The hormones detected were Auxins, Cytokinins (CKs), Abscisic Acid (ABA), Jasmonates (JAs), Salicylic Acid (SA), Gibberellins (GAs), Ethylene (ETH), Strigolactones (SLs), Brassinolide (BR), and other hormone precursor substances and derivatives. The instrumentation system for endogenous hormone data acquisition consisted of the UPLC-ESI-MS/MS system (ExionLC™ AD, UPLC) and LC-MS/MS (QTRAP^®^ 6500+, MS/MS). Based on the standards and the quantitative analysis of the data detected by mass spectrometry, the amount of hormone in the sample (ng/g) was calculated using the formula C*V/1000/m, where C represents the concentration value (ng/mL) obtained by substituting the ratio of the product peak area in the sample into the standard curve, V represents the volume of the solution (μL), and m represents the mass of the sample weighed (g).

### Statistical analysis

Statistically significant differences were analysed using a one-way analysis of variance (ANOVA) followed by Duncan’s multiple comparison test. All calculations were performed using SPSS software (version 21; IBM, Armonk, NY, USA) and all results are expressed as the mean ± standard deviation (SD) of at least three independent biological replicates. Treatment means were separated by Duncan’s multiple range test at p-values <0.05.

## Results

### Characteristics of secondary flower development in chestnut

**Figure 1:**
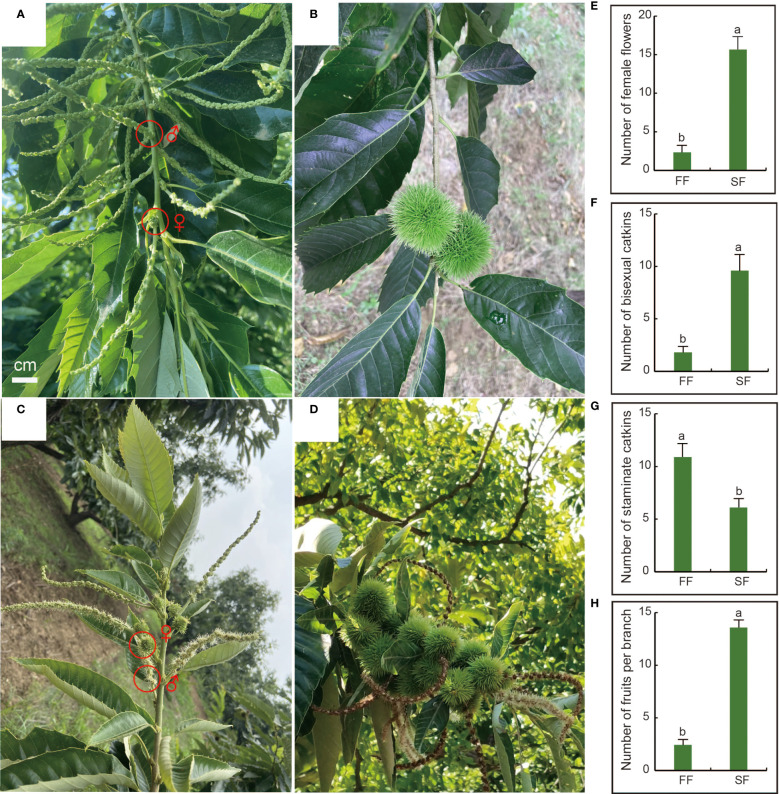
Differences in the number of male and female flowers between first and secondary flowering of chestnut. **(A)** Chestnut first flower morphology. The upper part of the branch bears a bisexual catkin, and the base of the bisexual catkin is a cluster of female flowers. **(B)** 1–2 fruits growing in the upper part of the first branch. **(C)** Secondary flowering form of chestnut. Bisexual catkin can be borne from the base to the top of the branch. **(D)** Secondary branches can bear fruit in bunches on the branches. **(E)** The number of female secondary flowers per fruiting branch is significantly higher than the number of female first flowers. **(F)** The number of bisexual inflorescences per fruiting branch is significantly higher than that of first flowers. **(G)** The number of first flowering staminate catkins per fruiting branch was significantly higher than that of secondary flowering. **(H)** The number of secondary fruits on each fruiting branch was significantly higher than that of first fruits. Letters show significant differences at *P* < 0.05 based on a t-test; error bars represent sd.

In northern China, chestnut trees are dormant in late autumn and November and start to sprout in late April. The normal flowering period is June (**Figure 1A**), and chestnut fruits ripen from late August to early October (**Figure 1B**). ‘Zunhuaduanci’ is a chestnut variety with secondary fruiting characteristics. It blooms and fruits again from August to October of the same year (**Figures 1C, D**), and the flowering and fruiting periods are shorter. In addition to the seasonal variation in chestnut flowering, we found that although the number of male flower clusters in secondary flowers was much higher than that of female flower clusters, the proportion of mixed inflorescences in secondary flowers was significantly higher, and the number of fruits was also significantly higher than that of the first fruit. In this study, we investigated the number of male and female flowers and the final number of fruit set during the two flowerings of ‘Zunhuaduanci’ from April to October. The results showed that, compared with the first flowering, the number of female flowers per fruiting branch increased significantly by 7-fold in the secondary flowering, reaching 15 (**Figure 1E**). The number of bisexual inflorescences increased significantly by 5-fold, reaching 9–10 (**Figure 1F**), and the number of male inflorescences per fruiting branch decreased significantly by five (**Figure 1G**). Further, the number of fruits on a single fruiting branch was approximately 14 after the second flowering, while the primary flowering had approximately two fruits per fruiting branch (**Figure 1H**). This phenomenon is beneficial for the development of a method to increase female flowers and reduce male flowers in chestnuts by exploring the formation of secondary flowers and analysing the reasons for the increase in the number of female flowers. This is of great significance for chestnut production.

### Transcriptome analysis between female flowers and male flowers in spring and late summer

**Figure 2:**
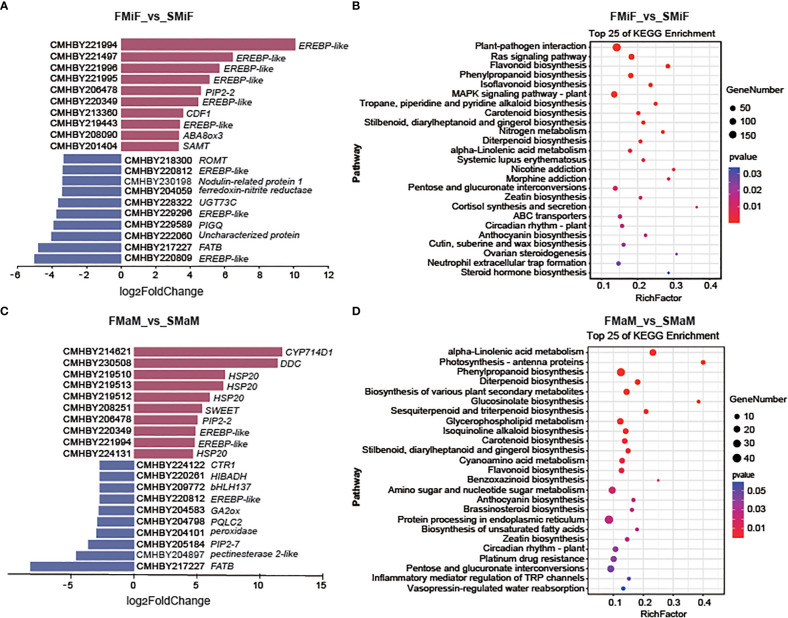
Transcriptome analysis of female and male flowers in different seasons. **(A)** Top 10 upregulated and downregulated DEGs between first and secondary female flowers. **(B)** KEGG enrichment of DEGs of FMiF_vs_SMiF. **(C)** Top 10 upregulated DEGs and downregulated DEGs between first and secondary male flowers. **(D)** KEGG enrichment of DEGs of FMaM_vs_SMaM. CDF1, cycling dof factors; *ABA8, abscisic acid 8’-hydroxylase; ROMT, trans-resveratrol di-O-methyltransferase; SAMT, salicylate 1-O-methyltransferase; UGT73C, UDP-glucosyl transferase; PIGQ, phosphatidylinositol N-acetylglucosaminyltransferase subunit Q; FATB, fatty acyl-ACP thioesterase B; CYP714D1, gibberellin 16alpha,17-epoxidase; SWEET, solute carrier family 50 (sugar transporter); CTR1, solute carrier family 31; HIBADH, 3-hydroxyisobutyrate dehydrogenase; GA2ox, gibberellin 2beta-dioxygenase; PQLC2, solute carrier family 66; FATB, fatty acyl-ACP thioesterase B.*

To investigate the cause of secondary flower formation in chestnuts, four cDNA libraries were constructed: first flowering mixed inflorescence basal female flower (FMiF), first flowering male inflorescence basal male flower (FMaM), secondary flowering mixed inflorescence basal female flower (SMiF) and secondary flowering male inflorescence basal male flower (SMaM), with three biological replicates for each sample (data unpublished). Pairwise comparisons of the four cDNA libraries (FMiF vs. SMiF and FMaM vs. SMaM) were used to identify DEGs between samples from different periods. Upregulated DEGs between female flowers were mainly EREBP-like transcription factors and others genes, including *cycling dof factors* (*CDF1*) and *abscisic acid 8’-hydroxylase* (*ABA8ox3*). Downregulated DEGs included several EREBP-like transcription factors such as *salicylate 1-O-methyltransferase* (*SAMT*), *gibberellin 2beta-dioxygenase* (*GA2ox*) and *fatty acyl-ACP thioesterase B* (*FATB*) (**Figure 2A**). KEGG pathway enrichment analysis of genes between female flowers showed that these genes were mainly enriched in Plant-pathogen interactions, the RAS signalling pathway, Flavonoid biosynthesis, Phenylpropanoid biosynthesis, and Carotenoid biosynthesis (**Figure 2B**). The upregulated genes in male flowers included *HSP20* and some EREBP-like transcription factors, and their downregulated genes included *EREBP-like*, *GA2ox*, and *FATB* (**Figure 2C**). KEGG enrichment analysis showed that the DEGs between male flowers were mainly enriched in the pathways for Alpha-linolenic acid metabolism, Photosynthesis-antenna proteins, Phenylpropanoid biosynthesis, Glucosinolate biosynthesis, Carotenoid biosynthesis, and Flavonoid biosynthesis (**Figure 2D**).

**Figure 3:**
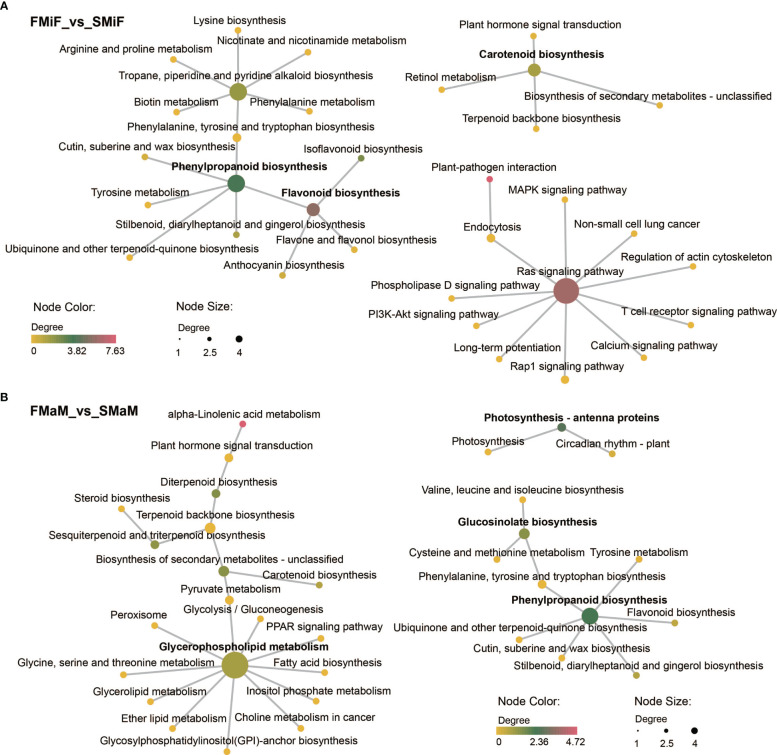
KEGG core pathway relationships. **(A)** KEGG network diagram between FMiF_vs_SMiF. **(B)** KEGG network diagram between FMaM_vs_SMaM. Different nodes indicate the KEGG pathway, and the colour and size of the nodes indicate the degree of KEGG enrichment. The section in bold represents the core KEGG pathway.

KEGG network diagrams are often used to represent interactions between enriched pathways, discover upstream and downstream signalling pathways, and find core pathways. We performed a correlation analysis of KEGG enrichment pathways in the comparison between FMiF vs. SMiF and FMaM vs. SMaM groups. The KEGG pathways between female flowers were divided into three clusters, with the core pathways being Phenylpropanoid biosynthesis, the RAS signalling pathway, and Carotenoid biosynthesis. Phenylpropanoid biosynthesis was linked to Flavonoid biosynthesis, whereas Carotenoid biosynthesis was correlated with Plant hormone signal transduction (**Figure 3A**). The KEGG pathways between male flowers were also divided into three clusters, with the core pathways being Glycerophospholipid metabolism, Phenylpropanoid biosynthesis, and Photosynthesis-antenna proteins, which are directly related to Fatty acid biosynthesis, Flavonoid biosynthesis, and Circadian rhythm-plant and Photosynthesis-antenna proteins, respectively (**Figure 3B**). In summary, we suggest that secondary flower development mainly involves the Circadian rhythm-plant, Photosynthesis-antenna proteins, Phenylpropanoid biosynthesis, Carotenoid biosynthesis, and Plant hormone signal transduction. Among these, *EREBP-like* and *HSP20* were the main regulatory genes.

**Figure 4:**
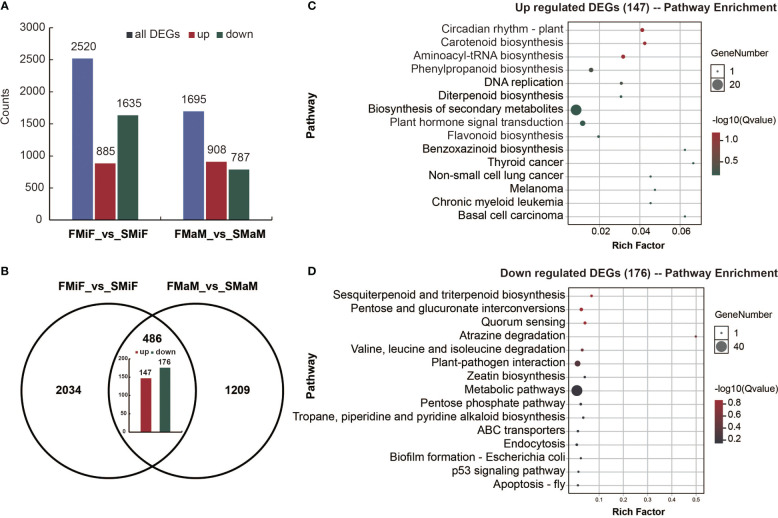
Transcriptome co-upregulation and co-downregulation analysis of secondary flowers compared with first flowers. **(A)** The statistical analysis of total, upregulated and downregulated DEGs in different comparison groups. **(B)** Venn diagram of DEGs obtained from FMiF _vs_SMiF and FMaM_vs_SMaM. **(C)** KEGG enrichment analysis 147 co-upregulated DEGs. **(D)** KEGG enrichment analysis of 176 co-downregulated DEGs.

To determine which DEGs had the same expression patterns between FMiF vs. SMiF and FMaM vs. SMaM. A total of 885 upregulated and 1635 downregulated DEGs were detected in FMiF vs. SMiF, and 908 upregulated and 787 downregulated DEGs were detected in FMaM vs. SMaM (**Figure 4A**). A total of 147 upregulated genes (**Table S1**) and 176 downregulated genes (**Table S2**) were identified in the two sets of comparisons (**Figure 4B**). We further performed KEGG enrichment analysis on the co-upregulated and downregulated genes and found that the co-upregulated genes were mainly concentrated in Circadian rhythm-plant, Carotenoid synthesis, Phenylpropanoid biosynthesis, Flavonoid biosynthesis, and Plant hormone signal transduction (**Figure 4C**), while the downregulated genes were mainly concentrated in Zeatin biosynthesis, Plant-pathogen interaction, and other pathways (**Figure 4D**). Genes with the same expression are more indicative of the causes of secondary flower formation in plants.

### Metabolome analysis between male and female flowers in the spring and late summer

**Figure 5:**
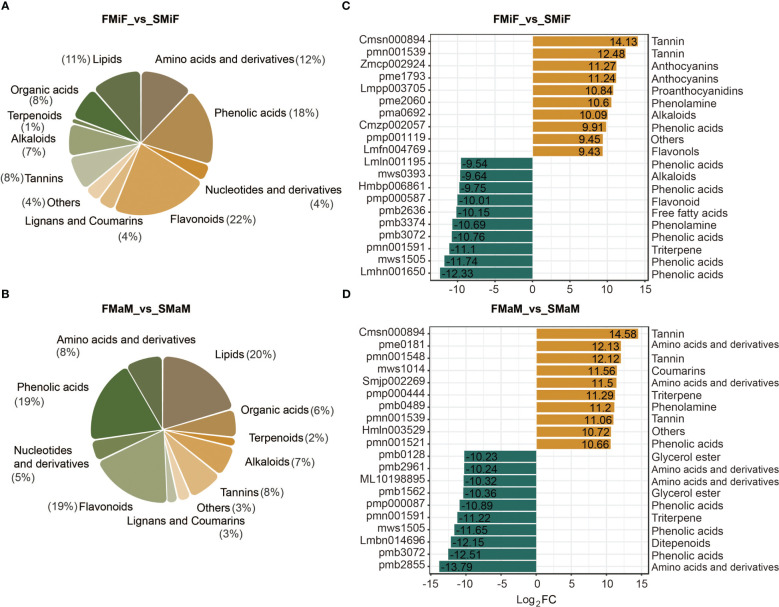
Analysis of metabolite differences between first and secondary flowers. **(A)** Metabolite species in FMiF_vs_SMiF. **(B)** Metabolite species in FMaM_vs_SMaM. **(C)** Differential metabolite upregulation and downregulation of the top 10 metabolites in FMiF_vs_SMiF. **(D)** Differential metabolite upregulation and downregulation of the top 10 metabolites in FMaM_vs_SMaM.

In total, 888 metabolites were analysed in chestnut flowers using UPLC-MS (unpublished data). The most differentially accumulated metabolites in FMiF vs. SMiF were flavonoids (22%) and phenolic acids (18%) (**Figure 5A**), whereas the differential metabolites in male flowers at different time points were mainly lipids (20%), followed by phenolic acids (19%) and flavonoids (19%) (**Figure 5B** and **Table S3**). We also analysed metabolite upregulation versus downregulation. The phenolic acid 6-O-acetylarbutin was upregulated in secondary flowering female flowers compared to first flowering female flowers, while upregulated flavonoids included cyanidin-3-O-(2’’-O-glucosyl) glucoside, Pelargonidin-3,5-O-diglucoside, and quercetin-3-O-(4’’-O-galloyl) arabinoside. Phenolic acids downregulated in secondary flowers included p-hydroxybenzoyltartaric acid, phenethyl caffeate, 3-O-p-coumaroyl shikimic acid-O-glucoside, 1,2-O-diferuloylglycerol, and picein (4-acetylphenyl-glucoside); the downregulated flavonoids were luteolin-7-O-(6’’-malonyl) glucoside (**Figure 5C**). Compared to first-flowering male flowers, secondary-flowering male flowers accumulated more tannin and phenolic acids, and peonidin-3-O-(6’’-O-malonyl) glucoside was a downregulated flavonoid (**Figure 5D** and **Table S4**).

**Figure 6:**
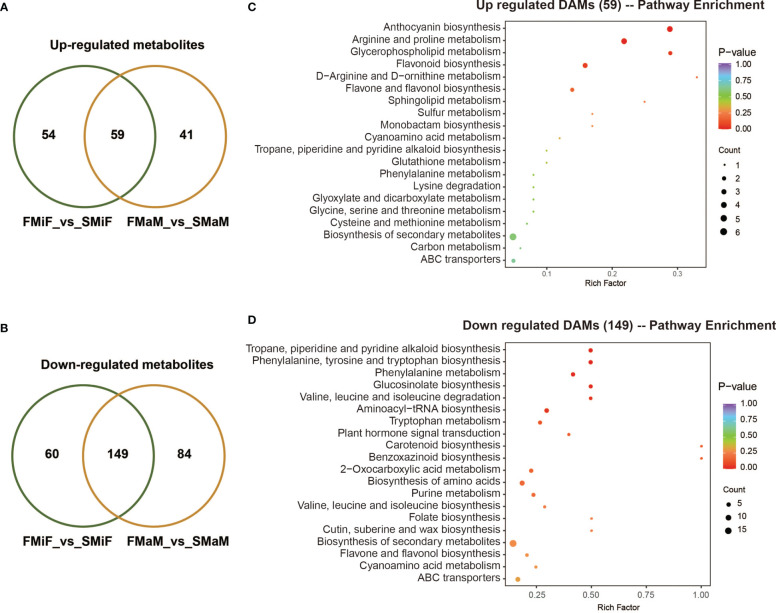
Analysis of co-upregulated and downregulated metabolites in first and secondary flowers. **(A)** A total of 59 metabolites were co-upregulated. **(B)** A total of 149 metabolites were co-upregulated. **(C)** KEGG enrichment analysis of 56 co-upregulated metabolites. **(D)** KEGG enrichment analysis of 149 co-downregulated metabolites.

Venn diagrams illustrate significantly different metabolites with the same expression pattern, with a total of 59 co-upregulated metabolites (**Figure 6A** and **Table S5**) and 149 co-downregulated metabolites (**Figure 6B** and **Table S6**) in both the FMiF vs. SMiF and FMaM vs. SMaM groups. To further elucidate the functions of these metabolites, we analysed their KEGG enrichment pathways. 59 co-upregulated DAMs were mainly enriched in anthocyanin synthesis, amino acid, lipid, and flavonoid synthesis in total (**Figure 6C**); in contrast to the transcriptome data, plant signalling pathways and carotenoid synthesis were present in the downregulated genes (**Figure 6D**).

### Hormonal analysis between male and female flowers in the spring and late summer

Because plant hormones have important effects on plant growth and development, we detected eight major hormone classes: auxins, CKs, ABA, JAs, SA, GAs, ETH, SLs, and BR (data not published). Analysis of hormones (**Tables 1**, **2**) showed that the Cytokinins IP and tZ, OPDA (a precursor substance of Jasmonic acid), MEJA (a Jasmonic acid derivative), and Gibberellin GA20 were significantly upregulated in secondary female flowers. In male flowers, 2MeScZR, MEJA, and tZ were significantly upregulated, whereas OPDA was significantly downregulated. Most hormones were in significantly lower amounts in secondary flowers than in first flowers. The ABA and SA contents in both female and male secondary flowers were significantly lower than those in first flowers, showing a more significant negative correlation. Other hormones that were jointly downregulated were the hormone derivatives SAG, ABA-GE, and BAP, which are not directly involved in plant hormone signal transduction. Therefore, it is speculated that these substances are involved in biosynthesis on the one hand, and can act directly or indirectly on the flowering genes to influence the flowering of chestnut on the other hand.

**Table 1:** Differences in hormone content in the comparison of FMiF_vs_SMiF.

Class	Index	FMiF ng/g FW	SMiF ng/g FW	Log2FC	Type
CK	IP	0.14	0.76	2.45	up
JA	OPDA	33.33	100.03	1.59	up
CK	tZ	0.65	1.71	1.40	up
JA	MEJA	2.52	5.49	1.12	up
GA	GA20	0.11	0.37	1.73	up
ABA	ABA	306	149	-1.04	down
GA	GA8	4.25	2.02	-1.07	down
JA	JA-Val	28.20	13.23	-1.09	down
Auxin	TRP	15366.67	5083.33	-1.60	down
SA	SA	844.67	276.00	-1.61	down
SA	SAG	1613.33	514.67	-1.65	down
ABA	ABA-GE	55.33	17.43	-1.67	down
JA	JA-ILE	454	129.67	-1.81	down
JA	H2JA	1.26	0.30	-2.07	down
CK	BAP	0.10	0.02	-2.19	down
CK	cZR	0.20	0.04	-2.42	down

**Table 2:** Differences in hormone content in the comparison of FMaM_vs_SMaM.

Class	Index	FMaM ng/g FW	SMaM ng/g FW	Log2FC	Type
CK	2MeScZR	2.23	7.31	1.71	up
JA	MEJA	2.23	6.14	1.46	up
CK	tZ	0.53	1.32	1.31	up
SA	SAG	1410	688.33	-1.03	down
ABA	ABA	240	116	-1.05	down
JA	OPDA	81.60	38.70	-1.08	down
ABA	ABA-GE	104.70	47.40	-1.14	down
CK	oT	0.33	0.15	-1.16	down
Auxin	IAA	14.07	6.09	-1.21	down
CK	cZR	0.09	0.04	-1.26	down
SA	SA	707	262.67	-1.43	down
CK	BAP	0.18	0.05	-1.82	down
Auxin	IAA-Asp	65.13	17.40	-1.90	down

### Integrated analysis of the relationship between the transcriptome and metabolome as well as phytohormones

**Figure 7:**
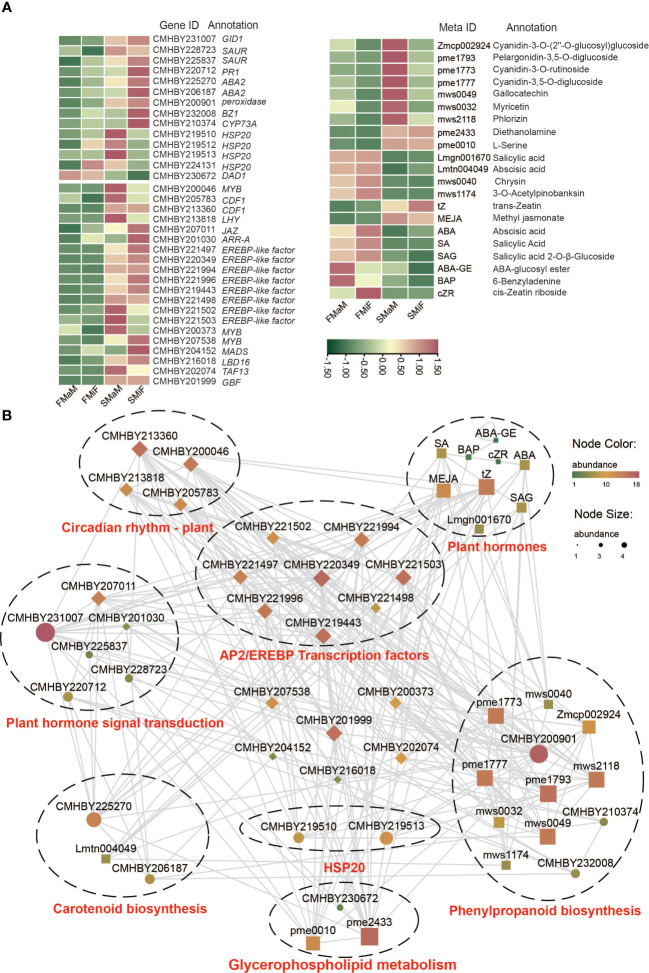
Correlation between DEGs, DAMs, and differential hormones of target pathways. **(A)** Heatmap of DEGs, transcription factors, DAMs and differential hormones. **(B)** Positive correlation between target genes and target metabolites. The node colour and size indicate the degree of connectivity, with larger nodes and redder colours indicating stronger connectivity between the two.

Association analysis of each histology can directly demonstrate the intrinsic regulatory mechanisms through network diagrams. Finally, we combined the Circadian rhythm-plant, plant hormone signal transduction, carotenoid biosynthesis, glycerophospholipid metabolism, arginine and proline metabolism, phenylpropanoid biosynthesis, and aminoacyl-tRNA biosynthesis as the target differential pathways for flower development during different chestnut periods. Based on these processes, the Pearson correlation coefficients between the two were calculated using differential genes, differential metabolites, and differential hormones associated with these pathways; EREBP-like transcription factors were screened in the transcriptome, and a positive correlation network graph and a negative correlation network graph were obtained. First, we plotted a heat map of the DEGs with differential metabolites (**Figure 7A**). It can be seen from the graph that all genes and transcription factors are significantly more expressed in secondary flowers than in first flowers, while there is an opposite trend for metabolites, especially for plant hormones such as ABA and SA. This suggests that they negatively regulate the formation of secondary flowers. Other metabolites showed the same gene expression patterns. Finally, we performed an association analysis using these metabolites with genes and screened *P* > 0.9 and *P* < −0.9 correlation coefficients for mapping (**Figure 7B** and **Table S7**). The results showed that the transcription factors involved in plant circadian rhythms were involved in the regulation of almost every pathway. It is also regulated by various pathways to alter the expression of *FT*, thereby affecting flowering. Flavonoids were the most diverse substances in the relational network, indicating their important roles in secondary flower development. ERF transcription factors are directly or indirectly involved in each pathway, and can be used as candidate genes to analyse secondary flowering. The temperature of secondary flowering was significantly higher than that of primary flowering, hence *HSP* expression was significantly increased under heat stress induction, thus improving the heat tolerance of chestnut during summer flowering. This can also be involved in promoting secondary flowering of chestnut together with ERF and the Flavonoid synthesis pathway, Circadian rhythm-plant, and Lipid synthesis pathway gene metabolites. In contrast, ABA and SA negatively correlated with most genes and metabolites (**Figure S1**).

### Myb-related protein 305 improves the male-to-female ratio of chestnuts by regulating flavonoid synthesis

**Figure 8:**
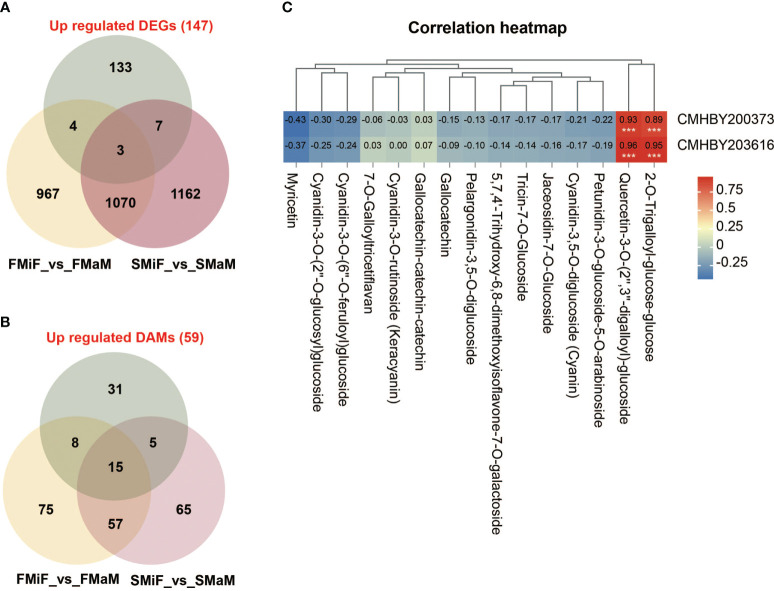
MYB305 transcription factor and flavonoids are involved in sexual differentiation of chestnut. **(A)** A Venn diagram of the association between 147 co-upregulated genes and male and female flower sex differences. **(B)** A Venn diagram of the association between 59 co-upregulated metabolites and male and female flower sex differences. **(C)** Heat map of the correlation between 2 genes and 15 flavonoid metabolites. Among them, *CMHBY204070* gene was excluded due to FPKM value <5.

Based on the above results, we identified the key pathways affecting secondary flower development in chestnuts. However, another obvious phenomenon of secondary flowering was that the number of female and bisexual flowers was significantly higher than that of first flowers. Therefore, we performed a Venn diagram analysis of upregulated DEGs (147) with FMiF vs. FMaM and SMiF vs. SMaM. We also analysed the upregulated DAMs (59) with FMiF vs. FMaM and SMiF vs. SMaM for metabolite differences, hoping that these results could predict the genes and metabolites that affect chestnut sex differentiation. Finally, we screened three genes, namely, *CMHBY204070*, *CMHBY200373* (*MYB305*), and *CMHBY203616* (solute carrier family 15, *SLC15*) and 15 metabolites (**Figure 8A**). However, the CMHBY204070 gene was not included in the subsequent analysis because all FPKM values were <5. All the 15 metabolites were flavonoids and their derivatives (**Figure 8B**). We correlated the two candidate genes with 15 metabolites, and the results showed that *MYB305* and *SLC15* were significantly and positively correlated with 2-O-trigalloyl-glucose-glucose and Quercetin-3-O-(2’’,3’’-digalloyl)-glucoside, respectively. In summary, we have developed a preliminary hypothetical model (**Figure 8C**). Because the timing of first and secondary flower formation in chestnuts is seasonal, it is likely that photoperiod and temperature are the main causes of secondary flower formation in chestnuts. LHY and CDF1 can interact with EREBP-like factors with *HSP* genes and are involved in the metabolic regulation of other pathways. Carotenoid biosynthesis synthesises ABA and phenylpropanoid biosynthesis synthesises SA, both of which can also respond directly to the external environment. Genes in the hormone signalling pathway interact with each other and with each transcription factor to participate in the synthesis of other flavonoid metabolites. In addition, we found that *MYB305* can affect the secondary flowering of chestnuts and may be a candidate gene affecting the sex differentiation of chestnuts, regulating the synthesis of flavonoids, and affecting the sex ratio of chestnut flowers.

## Discussion

In non-wood product forests, flowering plays an important role in the life cycle of plants, and the number of female flowers directly determines the final economic yield. As chestnuts are an important woody grain, the number of male flowers is much higher than that of female flowers during the growth process, resulting in a significant waste of resources. In some varieties with secondary flowering, the second flowering occurs in late summer and early autumn and the second wastes nutrients to avoid the formation of secondary flowers. However, the ratio of female to male flowers per fruiting branch in the secondary flowering of ‘Zunhuaduanci’ is significantly higher than that of the first flowering, which is an important material for our study on the sex differentiation of chestnut. In this study, we conducted a comprehensive analysis of the transcriptome, metabolome, and phytohormones involved in the formation of chestnut first and secondary flowers and comprehensively studied the developmental differences between chestnut secondary and first flowers. This provides a theoretical basis for preventing secondary flower formation and explores potential methods to improve the female-to-male chestnut ratio.

**Figure 9:**
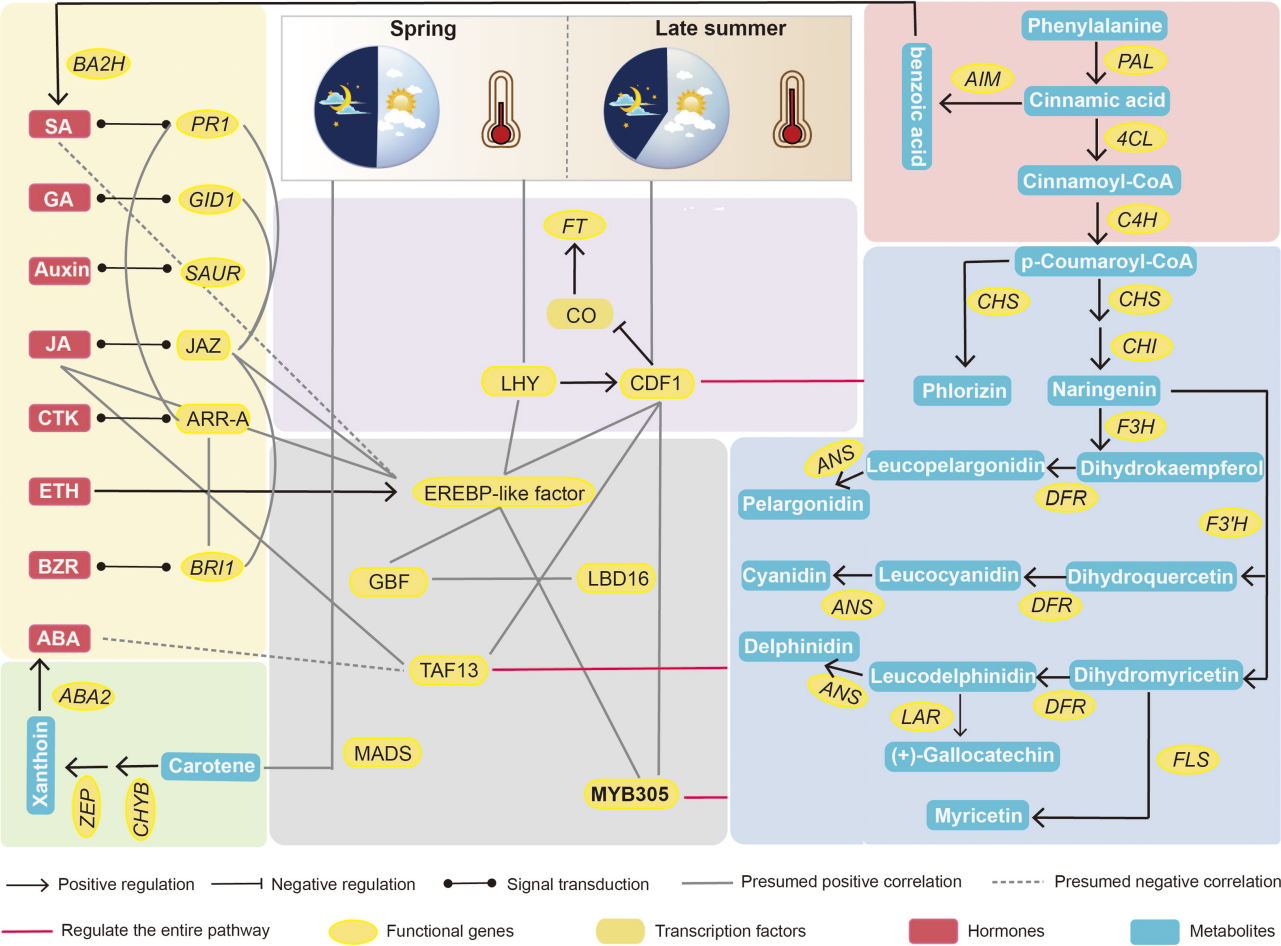
Integrated transcriptome, metabolome and phytohormones analysis provides new insights into the molecular regulatory network of the difference between first and secondary flower development in chestnut. MYB305 may be a key transcription factor affecting sex differentiation in chestnut.

The transition from flower formation to flowering in fruit trees is a complex physiological, biochemical, and morphogenetic process. Under certain conditions, plants receive environmental signals to produce signalling substances that are transported to the stem-end meristem to initiate flowering genes and transform the stem-end meristem into an inflorescence or floral meristem (Arkhimandritova et al., 2020). Temperate fruit trees often experience long winters before gaining the ability blooming (Luo and He, 2020). In the present study, the developmental differences between the secondary and first flowers of chestnuts were revealed using integrated transcriptomic, metabolomic, and phytohormonal analyses. EREBP-like was the most significantly upregulated gene in the female flowers (**Figure 2A**). *APETALA2/EREBP-like* genes constitute a large family of plant TFs that are involved in plant growth, development, and stress responses (Zeng et al., 2016). Overexpression of *Chrysanthemum* CmERF110 in *Arabidopsis* affects the photoperiodic pathway of *Arabidopsis* with an earlier flowering time (Xing et al., 2019). Rice *LATE FLOWERING SEMI-DWARF* (*LFS*) encodes an AP2/ERF transcription factor that can respond to plant circadian rhythms to promote flowering in rice under long daylight conditions (Shim et al., 2022). In our study, there was also a higher expression of *CDF1* in secondary flowers than in first-flowering female flowers. The final model (**Figure 9**) also demonstrated that EREBP-like proteins interacted with *CDF1*. During biological clock regulation in plants, GIGANTEA (GI) activates the expression of the key floral regulators *CO* and *FT* by degrading the CYCLING DOF FACTOR (CDF) to promote flowering (Fornara et al., 2015). In contrast, *HSP20* appeared to affect flower development more strongly in male flowers (**Figure 2C**). The development of male organs in plants is highly sensitive to temperature, and sustained high temperatures can cause male sterility (Djanaguiraman et al., 2018). High temperature stress in soybeans induces the upregulation of heat shock factor (HSF), ethylene-responsive TF RAP2-2 (ER RAP2-2), *MYB305*, and other genes that increase the tolerance of male soybean flowers to high temperatures (Ding et al., 2020). This is similar to our results, indicating that the late summer photoperiod and high temperature affected the secondary flowering of chestnuts, and that the expression of *EREBP* and *HSP* promotion played a protective role in secondary flowering. Temperature is also an important timing factor for biological clocks and certain *HSP* genes are controlled by circadian rhythms (Rikin, 1992). Our results suggest that a strong correlation between *HSP* genes and circadian rhythms exists, which warrants further investigation on their interactions in different temperature environments.

Endogenous plant metabolites also affect plant flowering. ABA is considered a flowering inhibitor, and ABSCISIC ACID-INSENSITIVE 4 (ABI4) is a key pathway in ABA signalling that inhibits flowering by directly binding to *FLOWERING LOCUS C* (*FLC*) transcription (Shu et al., 2016). Abscisic acid can also promote dormancy in fruit tree buds during cold winter (Li et al., 2018; Yang et al., 2020). However, it has also been demonstrated that ABA can respond to the photoperiod and plant biological clock to set off the flowering gene network and eventually induce the expression of *CO* and *FT* to trigger flowering (Kavi Kishor et al., 2022). SA can induce plant flowering in response to stress, and SA treatment advances the flowering time of saffron (Banday and Nandi, 2015; Rastegari et al., 2022). Because SA synthesis is derived from the phenylpropane pathway, the induction of flowering is accompanied by an increase in phenylalanine ammonia-lyase (PAL) activity (Wada and Takeno, 2010). The regulation of flower development by plant hormones is always accompanied by a complex network of crosstalk between hormones, which is induced by the light environment and can trigger stress in the external environment (Van de Poel et al., 2015). However, in the present study, ABA negatively regulated secondary flower formation in response to SA (**Figure 7**). The hormonal regulation of flower opening is complex. This complexity was elucidated by the regulatory network identified using the transcriptome and metabolome (**Figures 3**, **9**). In this network, carotenoid and phenylpropanoid biosynthesis can detect changes in plant circadian rhythms. As a synthetic precursor of ABA, LHY directly inhibits 9-cis-epoxycarotenoid dioxygenase enzymes in the flavonoid pathway (Adams et al., 2018). Enzyme genes that catalyse flavonoid metabolism, such as *CHS* and *F3′H*, can interact with the biological clock (Hildreth et al., 2022). components with a circadian rhythm (night light-inducible and clock-regulated), and RVE8 work together to regulate the anthocyanin metabolic pathway (Pérez-García et al., 2015). In combination with our metabolomic studies, flavonoids play an important role in the secondary flowering process (**Figures 5**, **6**). Summer heat stress affects lipid content (Lahlali et al., 2014) as a signalling metabolite for flowering time in Arabidopsis and oilseed rape, which can lead to earlier flowering through up-regulation of *FT* genes (Chakraborty et al., 2022). Our study also shows that lipids can be involved in other pathways to co-regulate secondary flowering in chestnut. Therefore, we believe that the cause of secondary flowering in chestnuts is related to the plant genotype. Not all varieties can form secondary flowers under the same geographical conditions; it may be due to the fact that secondary flowering varieties are more susceptible to stress because of their own weaker resistance. However, this could also be due to the abnormal flowering of plants caused by drought due to higher temperatures in late summer and early autumn. This is the result of changes in metabolites, especially flavonoids and hormones, in the body after external stress and changes in the circadian rhythm of the plant. However, the cause of the secondary flower opening in chestnuts requires further investigation.

In our previous study, we demonstrated that flavonoids could affect sex differentiation in chestnuts (unpublished data). In this experiment, by comparing the genes upregulated in secondary flowers relative to first flowers with the differential genes between male and female chestnut flowers, we screened *MYB305* with 15 flavonoid metabolites, further demonstrating the role of flavonoids in plant sex differentiation. In *Arabidopsis*, AtMYBD is involved in the circadian regulation of MYBL2 expression to promote anthocyanin accumulation (Nguyen and Lee, 2016). In conclusion, our experiments demonstrated that flavonoids are involved in sex expression in plants.

## Conclusion

Secondary flowering of fruit trees not only affects the next year’s yield, but also depletes tree nutrients, weakens the tree, and is very unfavourable for the overwintering of fruit trees. Therefore, for chestnut cultivation, it is important to increase the formation and opening of the first flowers while improving the male-to-female ratio. By analysing the development of first and secondary flowers in ‘Zunhuaduanci’, we found that the development of secondary flowers was mainly related to Circadian rhythm - plant, Plant hormone signal transduction, and Carotenoid biosynthesis, Glycerophospholipid metabolism, and Phenylpropanoid biosynthesis. These pathways interact and influence each other, and altering the genes or metabolites in these pathways may promote flower formation in chestnuts. We found that the transcription factor MYB305 could be a candidate gene that influences sex differentiation of plants by positively regulating flavonoid synthesis. However, the precise role of this protein requires further investigation. In conclusion, we constructed a regulatory network for secondary flower development in chestnuts, which provides a theoretical basis for the reproductive development mechanism of chestnuts and a new entry point for increasing chestnut yield and enhancing commercial value in production.

## Data availability statement

The data presented in the study are deposited in the ational Center for Biotechnology Information repository, accession number SRR21847692, SRR21847693, SRR21847682, SRR21847677, SRR21847678, SRR21847676, SRR21847675, SRR21847690, SRR21847691, SRR21847689, SRR21847687 and SRR21847688.

## Author contributions

SS, CQ, and TD conceptualized the study. CQ, TD, RZ, QW, YL, TW, HC, QB, and YZ performed the research. SS, CQ, and TD analyzed the data and wrote the paper with input from all authors. All authors contributed to the article and approved the submitted version.
