# Supplementary material for: Integrated transcriptome, metabolome and phytohormone analysis reveals developmental differences between the first and secondary flowering in Castanea mollissima
Source: Front Plant Sci. 2023 Mar 16;14:1145418. doi: 10.3389/fpls.2023.1145418 (PMC10060901; doi:10.3389/fpls.2023.1145418)
Supplement: Supplementary file 1 [file DataSheet_1.docx]

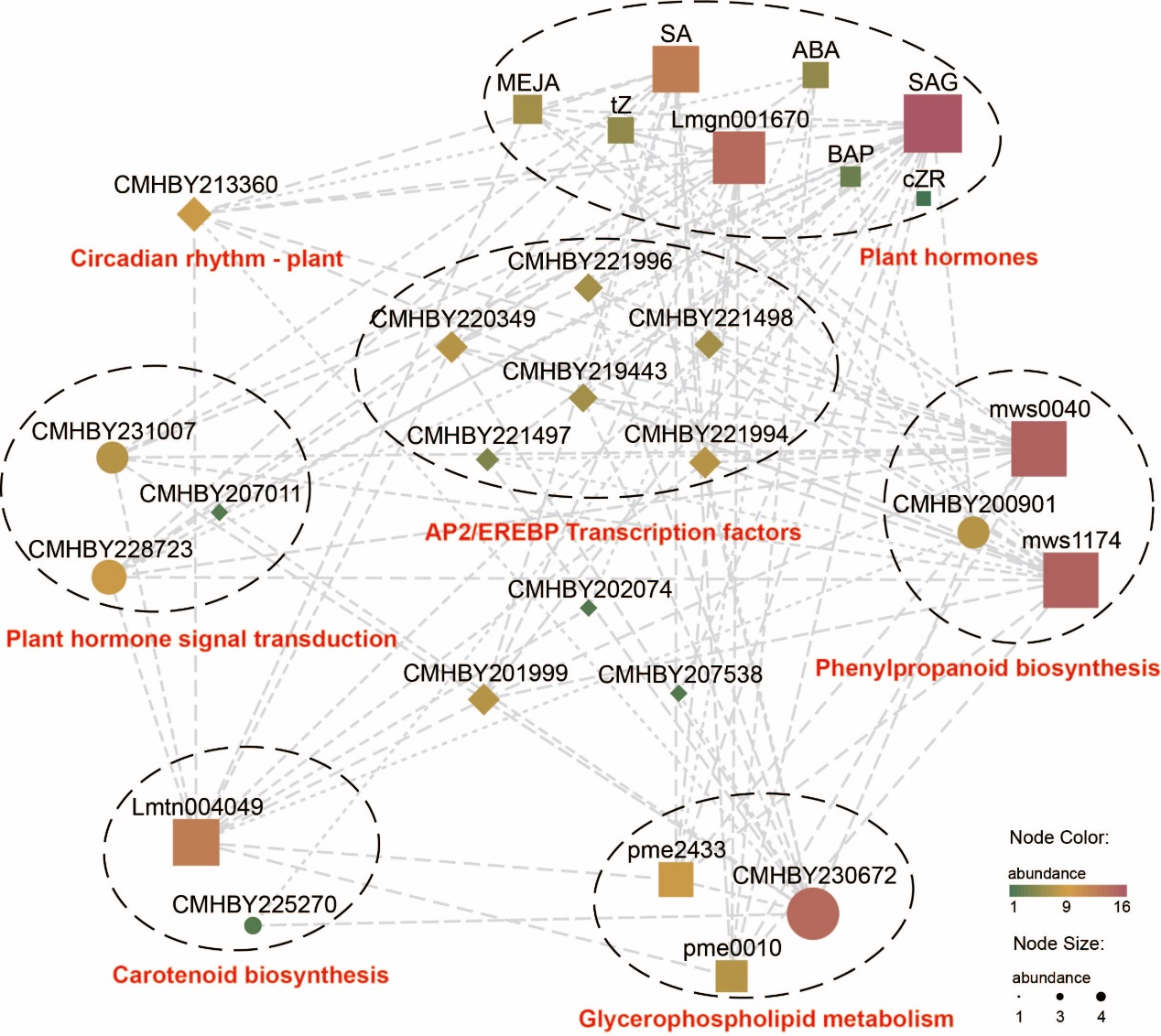


Supplementary Figure S1: Negative correlation between target genes and target metabolites. The node color and size indicate the degree of connectivity, with larger nodes and redder colors indicating stronger connectivity between the two.
